# Supplementary material for: Cassiosomes are stinging-cell structures in the mucus of the upside-down jellyfish Cassiopea xamachana
Source: Commun Biol. 2020 Feb 13;3:67. doi: 10.1038/s42003-020-0777-8 (PMC7018847; doi:10.1038/s42003-020-0777-8)
Supplement: Supplementary file 1 — Supplementary Information [file 42003_2020_777_MOESM1_ESM.pdf]

Supplementary Figures

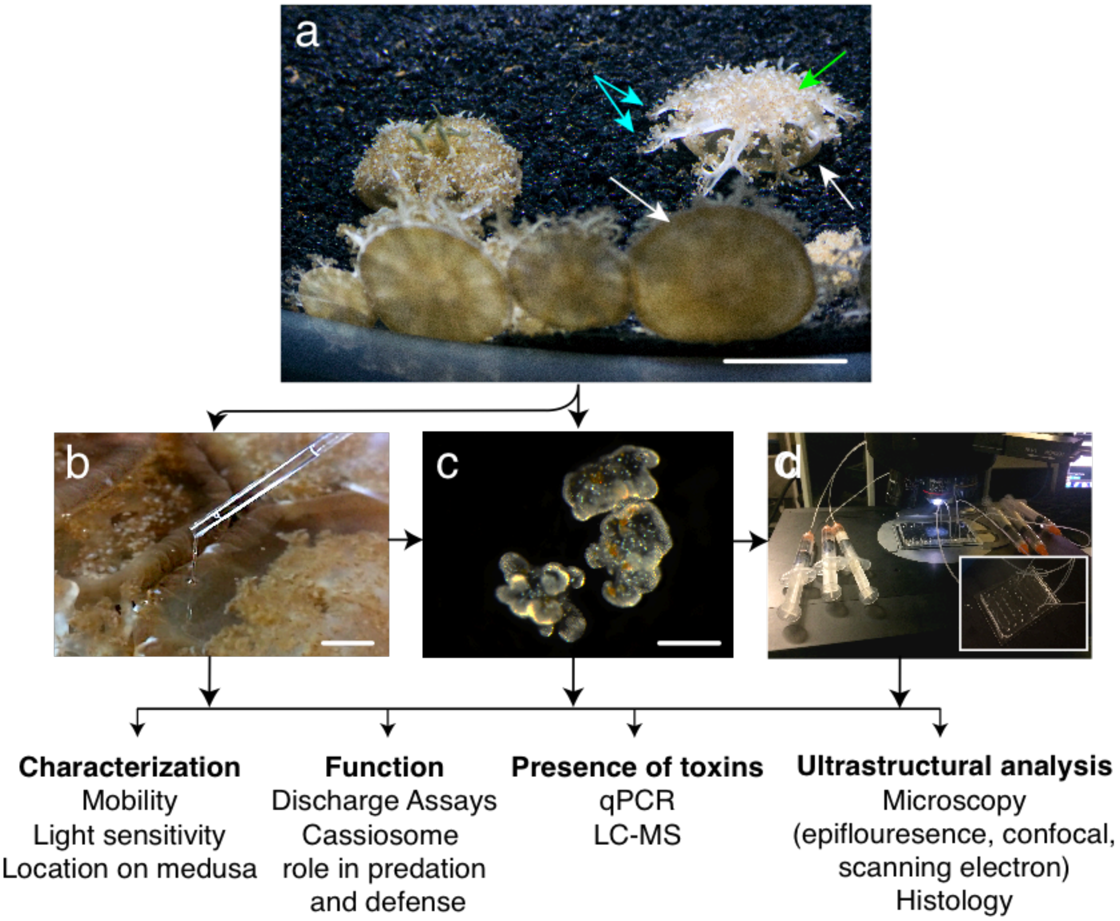

e

PRIMER NAME	PRIMER SEQUENCE (5'-3')	AMPLIFIED FRAGMENT LENGTH (BP)	TM VALUE	GC CONTENT
CTXC_QPCR_F1	5'-ACACAGAGGCAGCTTCATTTA-3'	284	62	42.9
CTXC_QPCR_R1	5'-ACTCTTTCCACCCTGACTTTG-3'	284	62	47.6
CTXC_QPCR_F2	5'-TGTAAGCGGAACACCAGTTAAT-3'	325	62	40.9
CTXC_QPCR_R2	5'-GCTGTAGCACTCATCAAGGTAA-3'	325	62	45.5
CTXC_QPCR_F3	5'-GGATGGCTACTGGATGATGAAA-3'	213	62	45.5
CTXC_QPCR_R3	5'-ACGCTATACCTGTTGGTAACAC-3'	213	62	45.5

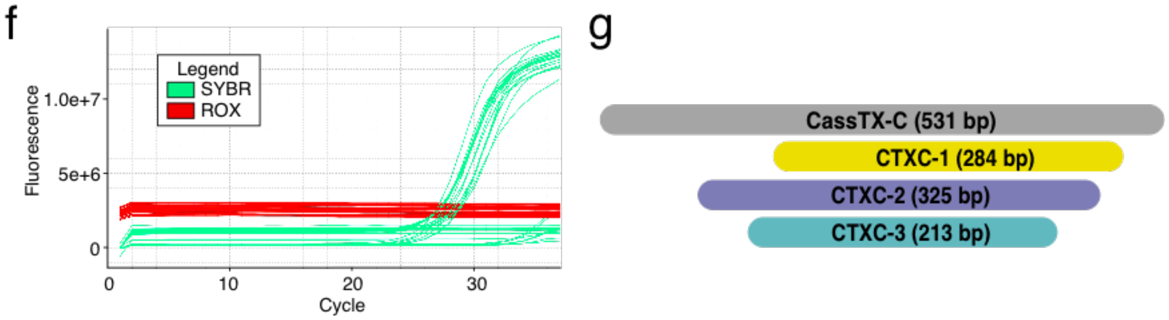

Supplementary Figure 1. Graphical abstract of cassiosome study workflow.

(a) *Cassiopea xamachana* medusae (approximately 5 – 10 cm diameter) photographed live in National Aquarium, Jellies Invasion Exhibit (Baltimore, USA), on substrate apex down or against the aquarium glass (orange arrows) brownish due to endosymbiotic *Symbiodinium* dinoflagellates in the umbrella); oral arms up (white arrows) bearing vesicular appendages (red arrows) appearing as white spots. (b) Mucus released from medusae is collected using a glass pipette. (c) Cassiosomes are isolated and examined within a microfluidic device (d). Microscopy is used for imaging, video-documentation and discharge assays. Direction of arrows follows workflow of experiments and assays conducted. e) Primer sequences used in qPCR experiment to amplify genetic regions encoding CassTX-C cnidarian toxin protein (Genbank BK010720). f) Presence/absence spectra for qPCR experiment using *Aurelia* and *Alatina alata* as off-target DNA samples. Amplification occurred for *C. xamachana* cassiosomes and tissue samples, but not for DNA from the other jellyfish species used in the assay. g) Schematic showing overlap of three amplified regions of CassTX-C for each of the three designed primer sets (see Methods). Scale bars, a = 5 cm, b = 1 cm, c = 200 μm.

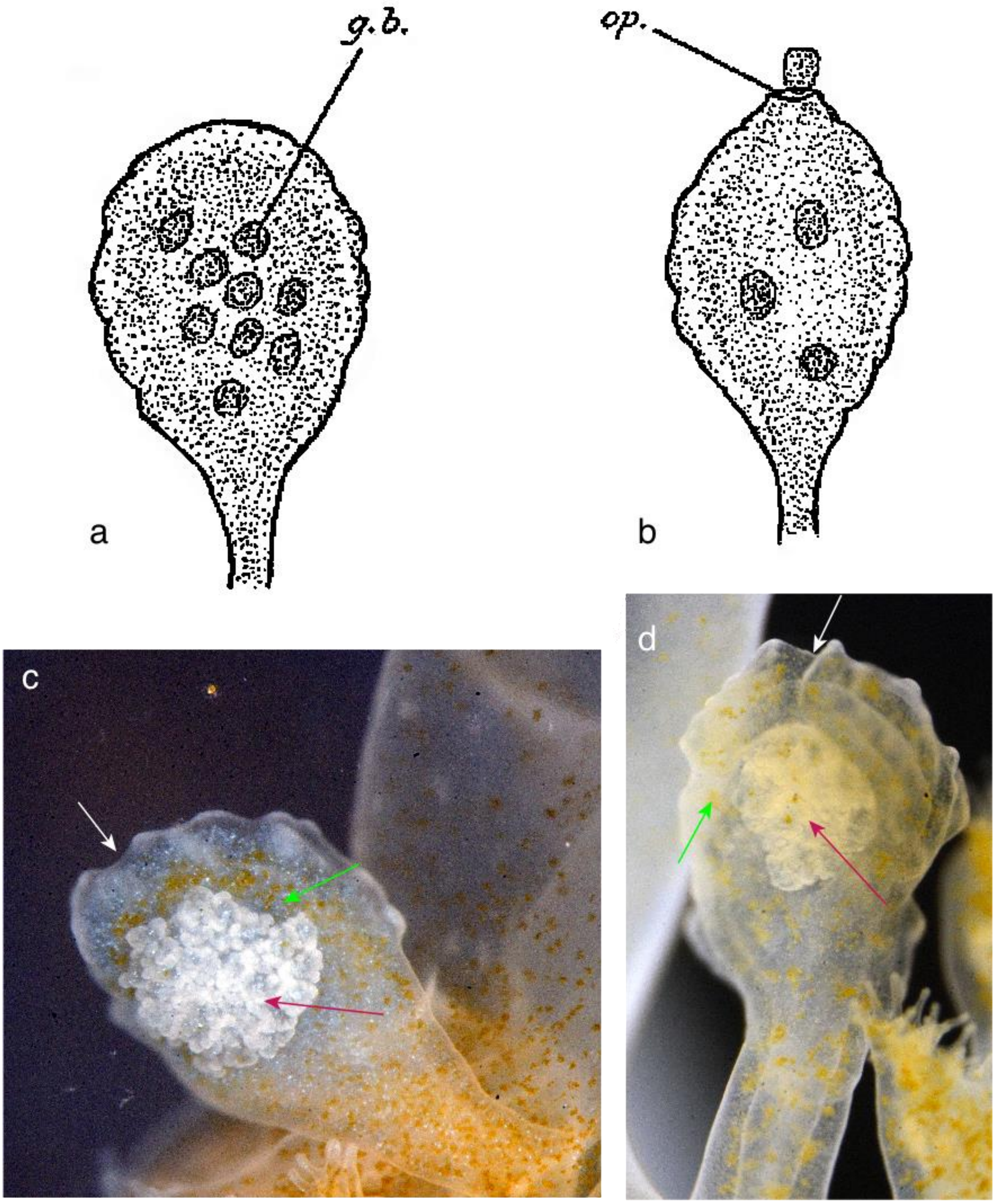

Supplementary Figure 2. Early report of cassiosome nests and detailed documentation in this study. (a,b) Line drawing of putative cassiosomes being released by *Cassiopea frondosa,* identified as “grey bodies” or a “nematocyst mass” through an apparent opening in the vesicular appendage. Image modified from Smith 1936 [Smith, H. G. Contribution to the anatomy and physiology of *Cassiopea frondosa. Pap. from Tortugas Lab. Carnegie Inst.*

Washingt. 18–52 (1936)]. Abbreviations: g.b. = nematocyte mass and op. = opening at tip of "oral vesicles" (i.e., vesicular appendage). (c,d) Vesicular appendages (green arrows) of *C. xamachana* photographed in this study lacking so-called aperture (only a groove with no opening occurs at the tip - white arrow). In *C. xamachana*, cassiosomes are shed from loosely organized nests (pink arrows) and released within mucus.

Database hit: CassTX-A (384 amino acids) **Genbank Accession: BK010718**

```
  1 MKDVLDEFTD EQLFKRMNSF VTTLGKIHGG IVASRKLSSD VEGNDLTEGD
 51 ITQLHTMIDL FSTDTLLGDV KQNIADEIIS EKVSDANRAL KTINVYCKLN
101 IVVELVLVEF INYIKEEGPE NNKLPTFYHS FMSTTRKNDK EYLGFLHLPE
151 VTEALVAAIY QNAPEKYPEL RQYIESIKVA PIPESGLEEG KTIYLTPKKW
201 PKWHFYLSSE SHSLIYGSTN TNDQNKFILK KASSGEEGRE WMIENKYYPN
251 YFISARKFES CLPLKHPDEV DYVEGLTMSY INRIKRNCGT QCLANGKCSG
301 CYSNCYSTFK IGRLRNLVSI NYVWRFTKLK SSGRCLYYFI SATQENFGPG
351 YTLFMKDSNN ANAYLKYGNP KEKGMFKLVK NSCK
```

Cassiosomes 198 → 51.6%

TIYLTPK QYIESIK IHGGIVASR (R)MNSFVTTLGK DSNNANAYLK QNIADEIISEK NLVSINYVWR RNCGTQCLANGK YYPNYFISAR **VAPIPESGLEEGK** (EEGPENNK)LPTFYHSFMSTTR DVLDEFTDEQLFK CSGCYSNCYSTFK ASSGEEGREWMIENK KLSSDVEGNDLTEGDITQLHTMIDLF**STDTLLGDVK**

Vesicular Appendages 195 → 50.7%

TIYLTPK QYIESIK MNSFVTTLGK DSNNANAYLK QNIADEIISEK(VSDANR) NLVSINYVWR YYPNYFISAR **VAPIPESGLEEGK** (EEGPENNK)LPTFYHSFMSTTR CSGCYSNCYSTFK DVLDEFTDEQLFKR KFESCLPLKHPDEVDYVEGLTMSYINR KLSSDVEGNDLTEGDITQLHTMIDLF**STDTLLGDVK**

Database hit: CassTX-B (304 amino acids) **Genbank Accession: BK010719**

```
  1 MRLLAVLCIL GFTLSNGRGD TTLDAETEAI FTQLETSLND KQQNKENQEE
 51 IKKLIQDVKD ELTKKDPEYG KKVLGMTKSL AGAVPKLKST NELTVAEGAL
101 LVVAGVAEHF PPPVGIVVAS LATLVSSVLG YLTPQKTNKA IKDAMTSVLN
151 DARDKDVKET LEGYQAELLT IKSYLAPKKK QTLDRDDVNN IVSNVNVHTG
201 ARELASLARR IQERAVSTDK NEAKRAFDFC VLYTKIATYR DAVLEEVIEL
251 FTKAGNTNEA ESYLNVKVTN IQQYKTALRF LHEPEASKAG ALVHYYPLGH
301 SKDS
```

Cassiosomes 84 → 27.6%

KKQTLDR DAMTSVLNDAR QQNKENQEEIK (K)LIQDVK(DELTKK) KVLGMTKSLAGAVPK DAVLEEVIELFTK AGNTNEAESYLNVK

Vesicular Appendages 84 → 27.6%

KKQTLDR DAMTSVLNDAR QQNKENQEEIK (K)LIQDVK(DELTKK) KVLGMTKSLAGAVPK DAVLEEVIELFTK AGNTNEAESYLNVK

Database hit: CassTX-C (176 amino acids) **Genbank Accession: BK010720**

```
  1 MSKIEEPMPW VAKKYVLRSV KWPSYHLERN RKKSKINLVK RYKNYMAFVS
 51 GTPVNATKIE FIPREDGYWM MKHRGSFIYA DDATQPTYTK VTRRAPAEDE
101 LGHWIVLKYF GKDLITISCR KWPDKFFNGV TNRYSVNLVD GNTDNGVQFY
151 LDECYSEEGA KSGWKEYNCP DFTPPE
```

Cassiosomes 16 → 9.1%

GSFIYADDATQPTYTK

Vesicular Appendages 30 → 17.0%

APAEDELGHWIVLK GSFIYADDATQPTYTK

Key:

Red: sequences exclusive to cassiosomes

Blue: sequences exclusive to vesicular appendages

Purple: sequences found in both samples

Black: sequences in predicted CassTX ORF (database hits) lacking spectral matches

Supplementary Figure 3. Peptide sequences detected in isolated cassiosomes and vesicular appendage tissues. Alignment of peptides identified via LC-MS/MS to *C. xamachana* toxin protein sequences. The protein sequence for each isoform of the cnidarian-restricted toxin (CassTX toxins -A, -B and -C) is shown. Peptides detected for each sample are shown below the protein sequence with color coding (red: only seen in the cassiosome sample; blue: only seen in the vesicular appendage sample; purple: seen in both the cassiosome and the vesicular appendage sample). The location of the peptides on the toxin sequence is also highlighted and the percent coverage of these identified peptides is listed.

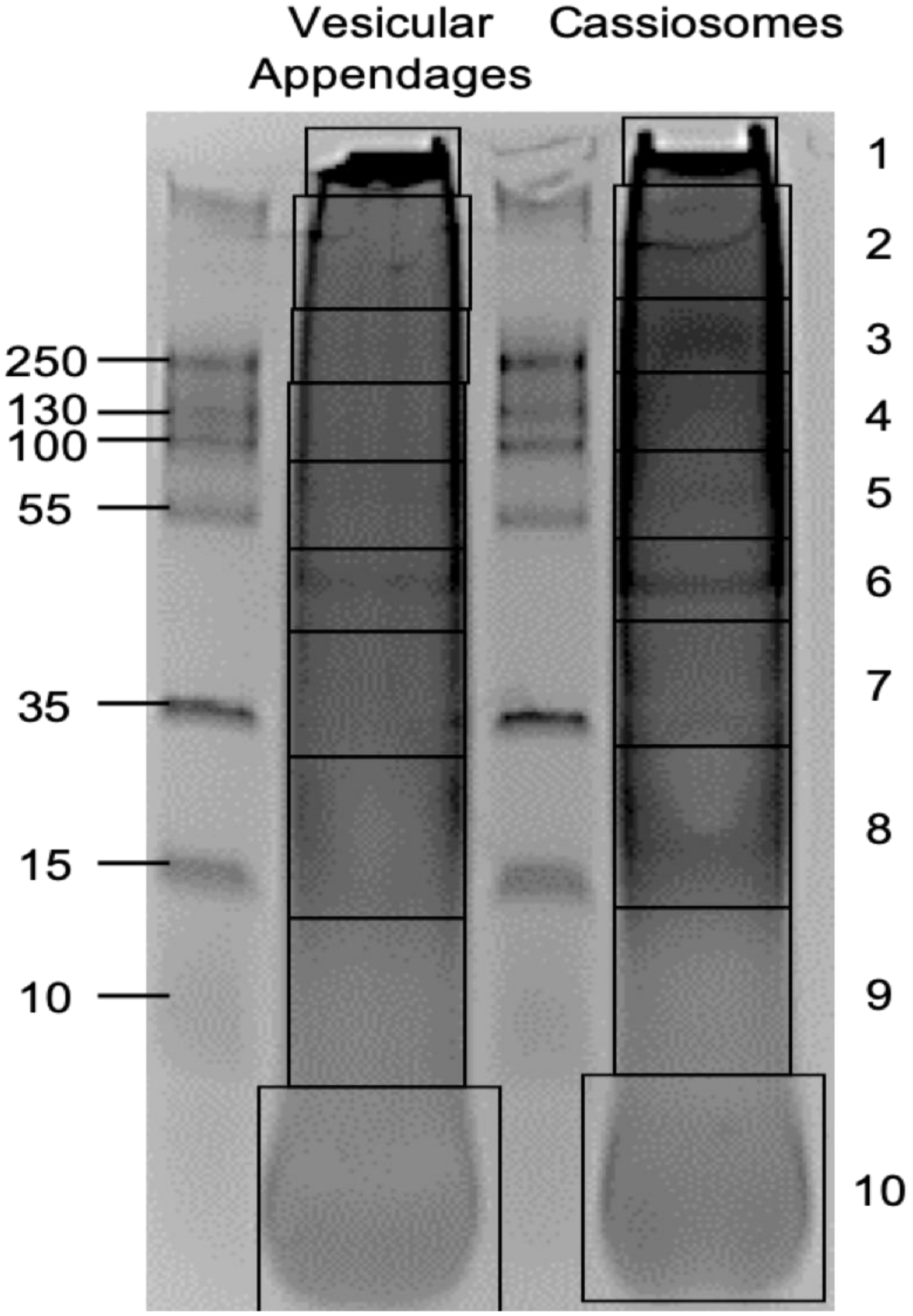

Supplementary Figure 4. SDS-PAGE gel of protein extractions for LC-MS/MS proteomic analysis from isolated cassiosomes and vesicular appendages. Ten bands were excised from the gel according to molecular weight. Results shown in Supplementary Figure 3 and Supplementary Table 2.

Supplementary Tables

**Tissue (diameter)**	**Nematocyst Type**	**Total**	**Length (∝m)** min-***mean***-max	**SD**	**Width (∝m)** **Min-*mean*-max**	**SD**
Polyp, infected	o-isorhiza	0	-	-	-	-
(n=2)	a-isorhiza	69	4.24-***6.17***-7.73	0.91	2.84-***3.84***-4.63	0.44
	Rhopalid	86	5.99-***9.33***-12.3	1.20	3.56-***7.20***-9.80	0.85
	Ls rhopalid	0	-	-	-	-
Polyp, uninfected	o-isorhiza	0	-	-	-	-
	a-isorhiza	35	5.39-***6.42***-7.60	0.68	3.09-***3.90***-4.82	0.35
	Rhopalid	37	7.54-***9.37***-11.6	1.08	5.94-***7.24***-8.59	0.73
	Ls rhopalid	0	-	-	-	-
Strobila (n=1)	o-isorhiza	3	6.41-***6.75***-6.97	0.30	5.98-***6.28***-6.73	0.40
	a-isorhiza	24	4.33-***5.15***-6.08	0.42	2.75-***3.42***-3.93	0.33
	Rhopalid	60	5.92-***9.23***-12.2	1.33	5.47-***7.31***-8.86	0.71
	Ls rhopalid	1	***7.68***	-	**5.31**	-
Ephyra (n=2)	o-isorhiza	26	4.74-***6.85***-8.31	0.92	4.45-***6.17***-7.45	0.80
	a-isorhiza	158	2.77-***5.04***-6.73	0.71	2.42-***3.37***-4.76	0.43
	Rhopalid	95	6.26-***9.22***-11.6	0.98	2.54-***7.54***-9.29	0.91
	Ls rhopalid	0	-	-	-	-
Medusa, small	o-isorhiza	25	5.31-***6.67***-8.29	0.84	4.96-***5.60***-6.96	0.53
(2.4 cm)	a-isorhiza	12	3.76-***5.06***-5.88	0.61	2.86-***3.10***-3.84	0.32
	Rhopalid	35	6.13-***9.41***-14.1	2.47	5.13-***7.26***-10.8	1.69
	Ls rhopalid	0	-	-	-	-
Medusa, medium	o-isorhiza	11	1.36-***6.87***-8.44	1.91	1.07-***5.76***-8.27	1.80
(6.4 cm)	a-isorhiza	23	1.45-***5.61***-8.30	1.24	1.17-***3.55***-4.13	0.55
	Rhopalid	56	7.15-***12.2***-14.8	2.38	6.46-***9.51***-13.0	1.76
	Ls rhopalid	3	6.70-***7.15***-8.06	0.79	4.1-***4.63***-5.19	0.54
Medusa, large	o-isorhiza	10	4.18-***6.97***-8.34	1.15	3.87-***5.73***-6.68	0.91
(8.8 cm)	a-isorhiza	11	4.23-***5.30***-7.44	0.82	2.56-***3.64***-5.30	0.70
	Rhopalid	27	8.59-***14.2***-16.4	1.98	6.12-***10.9***-12.4	1.62
	Ls rhopalid	0	-	-	-	-
Cassiosome	o-isorhiza	43	4.53-***6.80***-8.46	1.33	3.60-***5.58***-6.76	1.04
(n=4)	a-isorhiza	0	-	-	-	-
	Rhopalid	0	-	-	-	-
	Ls rhopalid	0	-	-	-	-
Mucus	o-isorhiza	1	***5.13***	-	***4.31***	-
	a-isorhiza	2	2.49-***3.46***-4.43	1.37	1.73-***2.55***-3.37	1.16
	Rhopalid	31	6.83-***8.98***-15.9	1.95	5.46-***6.83***-11.9	1.34
	Ls rhopalid	3	3.42-***4.20***-5.13	1.11	2.44-***3.02***-3.88	0.76
	Total	887				

Supplementary Table 1. Nematocyst measurements in C. xamachana cnidome using light microscopy. Measurements (average length and width) of undischarged nematocysts from homogenized C. xamachana tissue at multiple life stages (polyp, strobila/ephyra and medusa), and of cassiosomes and nematocysts (collected from medusae) suspended within mucus. Results presented in Figure 6.

Toxin	MW	Sample	Score	Spectra	Peptides	Coverage
CassTX-A	44.0 kD	Cassiosomes	8375	394	21	51.5%
		Vessicular Appendages	3447	184	17	50.7%
CassTX-B	33.5 kD	Cassiosomes	70	16	9	27.6%
		Vessicular Appendages	472	32	10	27.6%
CassTX-C	20.7 kD	Cassiosomes	26	1	1	9.1%
		Vessicular Appendages	118	5	2	17.0%

Supplementary Table 2. LC-MS/MS identification of cnidarian-restricted toxins. The results of mass spectrometry identification of the three toxin homologues (CassTX-A, -B, and -C). Score=Mascot probability score (assignment confidence of 0.05 > 60); Spectra=number of spectra assigned to the protein; Peptides=number of unique peptides identified in each sample type (cassiosomes or vessicular appendages; Coverage=percentage of protein sequence identified via unique peptides.
