# Supplementary material for: Cassiosomes are stinging-cell structures in the mucus of the upside-down jellyfish Cassiopea xamachana
Source: Commun Biol. 2020 Feb 13;3:67. doi: 10.1038/s42003-020-0777-8 (PMC7018847; doi:10.1038/s42003-020-0777-8)
Supplement: Supplementary file 2 — Description of Additional Supplementary Items [file 42003_2020_777_MOESM2_ESM.docx]

**Description of additional supplementary items**

**Supplementary Movie 1.** *Cassiopea xamachana* cassiosomes - various modes of motility. Random movement, central axis rotation, circular, and backing up (not shown here).

**Supplementary Movie 2.** *Cassiopea xamachana* discharge assay - cassiosomes. Isolated cassiosomes concentrated in a petri dish, killing numerous 1-day old *Artemia* nauplii within 60 sec.

**Supplementary Movie 3.** *Cassiopea xamachana* discharge assay - mucus only. Mucus concentrated in a petri dish, following manual removal of cassiosomes, subduing and trapping, but not killing, numerous one-day old *Artemia* nauplii within 60 sec.

**Supplementary Movie 4.** *Cassiopea xamachana* discharge assay – filtered artificial seawater control. Filtered artificial seawater used as a control for discharge assays. One-day old brine shrimp were exposed to water placed in a petri dish.

**Supplementary Movie 5.** Brine shrimp in microfluidic device. Isolated *Cassiopea xamachana* cassiosomes within a single chamber of a microfluidic device^41^ subdue, and then kill two-day old *Artemia* when introduced into the chamber.
