# Supplementary material for: Cassiosomes are stinging-cell structures in the mucus of the upside-down jellyfish Cassiopea xamachana
Source: Commun Biol. 2020 Feb 13;3:67. doi: 10.1038/s42003-020-0777-8 (PMC7018847; doi:10.1038/s42003-020-0777-8)
Supplement: Supplementary file 8 — Reporting Summary [file 42003_2020_777_MOESM8_ESM.pdf]

nature research

Corresponding author(s): Gary Vora, Cheryl Ames

Last updated by author(s): 2019/12/08

Reporting Summary

Nature Research wishes to improve the reproducibility of the work that we publish. This form provides structure for consistency and transparency in reporting. For further information on Nature Research policies, see Authors & Referees and the Editorial Policy Checklist.

Statistics

For all statistical analyses, confirm that the following items are present in the figure legend, table legend, main text, or Methods section.

n/a	Confirmed	
☐	☒	The exact sample size (*n*) for each experimental group/condition, given as a discrete number and unit of measurement
☐	☒	A statement on whether measurements were taken from distinct samples or whether the same sample was measured repeatedly
☐	☒	The statistical test(s) used AND whether they are one- or two-sided<br>*Only common tests should be described solely by name; describe more complex techniques in the Methods section.*
☒	☐	A description of all covariates tested
☒	☐	A description of any assumptions or corrections, such as tests of normality and adjustment for multiple comparisons
☒	☐	A full description of the statistical parameters including central tendency (e.g. means) or other basic estimates (e.g. regression coefficient) AND variation (e.g. standard deviation) or associated estimates of uncertainty (e.g. confidence intervals)
☒	☐	For null hypothesis testing, the test statistic (e.g. *F*, *t*, *r*) with confidence intervals, effect sizes, degrees of freedom and *P* value noted<br>*Give P values as exact values whenever suitable.*
☒	☐	For Bayesian analysis, information on the choice of priors and Markov chain Monte Carlo settings
☒	☐	For hierarchical and complex designs, identification of the appropriate level for tests and full reporting of outcomes
☒	☐	Estimates of effect sizes (e.g. Cohen's *d*, Pearson's *r*), indicating how they were calculated

Our web collection on statistics for biologists contains articles on many of the points above.

Software and code

Policy information about availability of computer code

Data collection	Nikon D7000 Camera using SharpShooter3 software<br>Olympus BX63 upright microscope, and viewed with cellSens software<br>Nikon A1R Confocal (Nikon Instruments) microscope, and viewed with NIS-Elements imaging software<br>Apreo FESEM (FEI) Scanning Electron Microscope<br>ViiA 7 Real-Time PCR System (ThermoFisher)<br>LC-MS/MS system (U3000 LC coupled to Orbitrap Fusion Lumos mass spectrometer (Thermo Scientific, Waltham, MA))
Data analysis	ImageJ software (Version 2.0.0-rc-68/1.52f, https://imagej.net)<br>NIS-Elements imaging software<br>BLAST+ (https://blast.ncbi.nlm.nih.gov/)<br>Transdecoder<br>Adobe Photoshop<br>Adobe Illustrator<br>PrimerQuest online software (http,//www.idtdna.com/PrimerQuest/)<br>Mascot (Matrix Science Inc., London, UK)<br>TransDecoder69 (v.5.5.0)<br>R base package (https,//www.R-project.org/) ggplot (http,//ggplot2.org)<br>iMovie<br>AMIRA ®

For manuscripts utilizing custom algorithms or software that are central to the research but not yet described in published literature, software must be made available to editors/reviewers. We strongly encourage code deposition in a community repository (e.g. GitHub). See the Nature Research guidelines for submitting code & software for further information.

Data

Policy information about availability of data

All manuscripts must include a data availability statement. This statement should provide the following information, where applicable:

- Accession codes, unique identifiers, or web links for publicly available datasets
- A list of figures that have associated raw data
- A description of any restrictions on data availability

The authors declare that all relevant data supporting the findings of this study are available within the manuscript and its Supplementary materials. Additional data are available from the corresponding authors upon request. The following datasets are publicly available:
1) Cassiopea xamachana transcriptome (PMCID: PMC5932825)
http://ryanlab.whitney.ufl.edu/downloads/Cnidaria_transcriptomes/
2) Cassiopea xamachana genome (NCBI Accession: OLMO00000000.1)
3) Cassiopea xamachana MS proteomics data (Figure 6; Supplementary Figure 3, 4, Supplementary Table 2) (PXD012177 and 10.6019/PXD012177): ProteomeXchange Consortium via the PRIDE partner repository70.
4) Cassiopea xamachana toxin protein GenBank accession numbers (Figure 5; Supplementary Figure 3) (NCBI Accession: BK010718, BK010719, BK010720).

Field-specific reporting

Please select the one below that is the best fit for your research. If you are not sure, read the appropriate sections before making your selection.

☐ Life sciences ☐ Behavioural & social sciences ☒ Ecological, evolutionary & environmental sciences

For a reference copy of the document with all sections, see nature.com/documents/nr-reporting-summary-flat.pdf

Ecological, evolutionary & environmental sciences study design

All studies must disclose on these points even when the disclosure is negative.

Study description	Snorkelers in mangrove forest waters inhabited by the upside-down jellyfish Cassiopea xamachana report discomfort due to a sensation known as “stinging water” or "toxic water", the cause of which was previously unknown. Using a combination of histology, microscopy, microfluidics, videography, molecular biology, and LC-MS/MS proteomics, we describe novel stinging-cell structures, which we term cassiosomes. These structures are released within C. xamachana mucus and are capable of killing prey. Cassiosomes consist of an outer epithelial layer mainly composed of nematocytes surrounding a core filled by endosymbiotic dinoflagellates hosted within amoebocytes and presumptive mesoglea. Furthermore, we report cassiosome structures in four additional jellyfish species in the same taxonomic group as C. xamachana (Class Scyphozoa; Order Rhizostomeae), categorized as either motile (ciliated) or nonmotile types. This inaugural study provides a qualitative assessment of the stinging contents of C. xamachana mucus and implicates mucus containing cassiosomes and free intact nematocytes as the cause of the “stinging water.”
Research sample	The upside-down mangrove jellyfish, genus Cassiopea (Phylum Cnidaria, Class Scyphozoa, Order Rhizostomeae) is among only a few rising model jellyfish taxa based on the recent release of the first draft genome. C. xamachana medusae, polyps, and ephyrae (juvenile medusae) of different sizes were examined in this study.
Sampling strategy	Most measurements in this study were conducted at least in triplicate (on tissue samples from at least three distinct life or tissue forms) for good measure, while videography and photodocumentation using light and confocal microscopy involved the use of hundreds to thousands of cassiosomes, validating our most important findings. The structure on the medusa in which the cassisomes are produced was also analyzed with histology.
Data collection	Data collection and analysis were conducted by AMLK, KM, and CLA; samples were prepared for LC-MS/MS by JS and CLA with substantial instruction by DL; JS conducted LC-MS/MS analysis with supervision by DL; microfluidic devices were designed and constructed by KB with substantial instruction by JR, who also provided training on their use for AMLK, KM, and CLA; SEM preparation and imaging was carried out by AR and CLA; fixation, histology, and imaging of semithins was done by AR; cassiosomes and vesicular appendage 3-D reconstruction was done by AR and NB; confocal microscopy was performed by MM, LDF and CLA; JDJ provided lab-reared rhizostome and Aurelia medusae, as well as Cassiopea polyps, which were reared to medusa stage by KM, MK, AMLK, CLA and AGC; JDJ assisted with mucus extraction from additional rhizostome species.
Timing and spatial scale	All data collection and analysis was conducted between July and September 2019 (at either Naval Research Laboratory or Smithsonian NMNH, Washington, D.C. USA, or National Aquarium, Baltimore) during which time coauthors were actively collaborating on the project.
Data exclusions	No data were excluded from analyses
Reproducibility	Experiments were conducted in triplicate (3 biological replicates at minimum) to ensure reproducibility. Detailed methods are provided.
Randomization	Samples were taken non-preferentially from different medusae (sometimes by different coauthors depending on the different methods employed). All rhizostome medusae examined in this study produced cassiosomes in their mucus. Medusae were preferentially selected when size comparison was important, e.g..., nematocyst measurements to characterize the C. xamachana

cnidome. Average length and width was measured of intact nematocysts from different tissues and life stages.(see Supplementary Table 1)

Blinding	Blinding was not relevant to the study, as all medusae examined produced cassiosomes. Also, it was important to know from which tissue type a sample was taken to validate and compare tissue types for certain analyses e.g., proteomics.

Did the study involve field work? ☐ Yes ☒ No

Reporting for specific materials, systems and methods

We require information from authors about some types of materials, experimental systems and methods used in many studies. Here, indicate whether each material, system or method listed is relevant to your study. If you are not sure if a list item applies to your research, read the appropriate section before selecting a response.

Materials & experimental systems

n/a	Involved in the study
☒	☐ Antibodies
☒	☐ Eukaryotic cell lines
☒	☐ Palaeontology
☒	☐ Animals and other organisms
☒	☐ Human research participants
☒	☐ Clinical data

Methods

n/a	Involved in the study
☒	☐ ChIP-seq
☒	☐ Flow cytometry
☒	☐ MRI-based neuroimaging
